# The use of routine outcome measures in two child and adolescent mental health services: a completed audit cycle

**DOI:** 10.1186/1471-244X-13-270

**Published:** 2013-10-20

**Authors:** Charlotte L Hall, Maria Moldavsky, Laurence Baldwin, Michael Marriott, Karen Newell, John Taylor, Kapil Sayal, Chris Hollis

**Affiliations:** 1 CLAHRC, University of Nottingham, Nottingham, UK; 2 CAMHS, Nottinghamshire Healthcare NHS Trust, Nottinghamshire, UK; 3 CAMHS, Derbyshire Healthcare NHS Foundation Trust, Derbyshire, UK; 4 Queens Medical Centre, University of Nottingham, Nottingham, UK; 5 B07 Institute of Mental Health, University of Nottingham, Triumph Road, NG7 2TU Nottingham, UK

**Keywords:** Routine Outcome Measurement (ROM), Child and Adolescent Mental Health Services (CAMHS), Audit

## Abstract

**Background:**

Routine outcome measurement (ROM) is important for assessing the clinical effectiveness of health services and for monitoring patient outcomes. Within Child and Adolescent Mental Health Services (CAMHS) in the UK the adoption of ROM in CAMHS has been supported by both national and local initiatives (such as government strategies, local commissioning policy, and research).

**Methods:**

With the aim of assessing how these policies and initiatives may have influenced the uptake of ROM within two different CAMHS we report the findings of two case-note audits: a baseline audit conducted in January 2011 and a re-audit conducted two years later in December 2012-February 2013.

**Results:**

The findings show an increase in both the single and repeated use of outcome measures from the time of the original audit, with repeated use (baseline and follow-up) of the Health of the Nation Outcome Scale for Children and Adolescents (HoNOSCA) scale increasing from 10% to 50% of cases. Re-audited case-notes contained more combined use of different outcome measures, with greater consensus on which measures to use. Outcome measures that were applicable across a wide range of clinical conditions were more likely to be used than symptom-specific measures, and measures that were completed by the clinician were found more often than measures completed by the service user.

**Conclusions:**

The findings show a substantial improvement in the use of outcome measures within CAMHS. These increases in use were found across different service organisations which were subject to different types of local service priorities and drivers.

## Background

Outcome measures offer a window on the clinical effectiveness of interventions, providing important information to clinicians, managers, commissioners and service users. The importance of measuring outcomes within Child and Adolescent Mental Health Services (CAMHS) was recognised in England through the government’s NHS Outcomes Framework policy [1] and the National Service Framework (NSF) [2] for children and young people. The NSF suggests that the effectiveness of CAMHS interventions should be evaluated from multiple perspectives, including the clinician, parent/carer and where possible, the young person. The NSF states that this information is vital to improve future clinical work and additional administrative, Information Technology (IT) and clinical support should be available to enable CAMHS to use outcome measures.

To help support the implementation of routine outcome measurement (ROM) within CAMHS, the CAMHS Outcome Research Consortium (CORC) was created. The Consortium originally recommended the use of four outcome measures: the Strengths and Difficulties Questionnaire (SDQ) [3], the Children’s Global Assessment Scale (C-GAS) [4], the Health of the Nation Outcome Scale for Children and Adolescents (HoNOSCA) [5], and the Commission for Health Improvement-Experience of Service Questionnaire (CHI-ESQ) [6]. More recently, CORC also developed a pilot measure, the Goals Based Outcome (GBO) [7] which enables the client to monitor goal achievement following an intervention.

The outcome measures advocated by CORC hold specific advantages. As 'generic’ outcome measures, they are applicable across a wide range of clinical conditions seen in child and adolescent psychiatry and can be used routinely regardless of the client’s condition. Data can be collated at a service level to provide important information to commissioners on the effectiveness of services, and can be used clinically to inform both the clinician and the service user of any changes that may result from the intervention. HoNOSCA and C-GAS can quantitatively provide the clinicians opinion of patient functioning, whereas the SDQ and GBO offer the service user voice on their functioning. The CHI-ESQ also provides the service user with an opportunity to comment on their experience of the services. According to the Consortium guidelines, the SDQ, HoNOSCA and C-GAS should be completed (as a minimum) at baseline (time 1) and 6-month follow-up (time 2). The CHI-ESQ should be completed upon discharge from the service. Measures that are completed at baseline can be important for informing the assessment process. However, measures that are only completed at one time point cannot measure within-individual change which is an essential feature of outcome measurement, as such the completion of the same measure at follow-up is crucial to meet the criteria of outcome measurement.

Since 2011, the Consortium has been commissioned by the Department of Health to support the analysis of outcome measurements collated through the Children and Young People’s Improving Access to Psychological Therapies (CYP-IAPT; http://www.IAPT.nhs.uk), an initiative created to improve services for families and young people attending CAMHS by routinely assessing their opinion on the quality and experience of services. The initiative also recommends the use of the SDQ, GBO and CHI-ESQ as outcome measures (http://www.iapt.nhs.uk/silo/files/cyp-iapt-outcomes-summary.pdf).

The CYP-IAPT recommends the completion of the SDQ and also an anxiety and depression scale (RCADS: Revised Child Anxiety and Depression Scale) [8] at initial assessment. At each subsequent session they suggest the use of brief scales such as the GBO and ORS (Outcome Rating Scale) [9] to measure functioning and the SRS (Session Rating Scale) [10] to assess client satisfaction. At the review stage the initiative advocates the use of the same measures that were completed at assessment (SDQ, RCADS) alongside a CHI-ESQ.

The research on implementing outcome measures in UK child and adolescent psychiatry indicates a relatively low uptake of these measures [11]. Johnston and Gowers’ [11] survey revealed that less than 30% of the 186 CAMHS teams that responded had routinely used ROM, with the SDQ and HoNOSCA being the most commonly used measures. Baruch and Vrouva [12] found a higher uptake of outcome measures for young people attending a psychotherapy unit where completion of self-reported measures was 94% at baseline, dropping to 35% at 3-months and even less at 6 and 12-month follow-up. In a recent multi-method study, Batty et al. [13] conducted a survey and workshop to assess stakeholders’ opinions of routine outcome measurement in CAMHS, and also carried out a case-note audit documenting the current use of outcome measures in three community CAMHS teams. In support of Johnston and Gowers [11] they also found a poor uptake of outcome measures.

Common reasons for the lack of completion of outcome measures in clinical practice reflect constraints on both time and resources [11,13-15]. The lack of timely feedback from completed outcome measures has also been shown to decrease clinicians’ motivation to use them, rendering them a 'tick box’ exercise used to meet targets but with little clinical utility [13]. Other noted barriers to the uptake of ROM include a lack of training [16,17], some clinicians unwillingness to use a systematic approach to data collection [18] and concerns about how the data will be used by managers and commissioners [19,20].

This study reports on the uptake of ROM across two CAMH services in the East Midlands region of England (Derbyshire and Nottinghamshire) and presents the findings of a re-audit and the baseline audit reported in Batty et al. [13]. Our aim is to provide an update of the current use of outcome measures and assess whether recent government strategies, (such as the increase in the provision of administration support to collect these measures) and local initiatives (such as the Commissioning for Quality and Innovation (CQUIN) target), alongside early CYP-IAPT trials and previous research has improved the uptake of outcome measures. This research was conducted as part of the National Institute for Health Research (NIHR) Collaborations for Leadership in Applied Health Research and Care- Nottinghamshire, Derbyshire and Lincolnshire (CLAHRC-NDL).

## Methods

Patient records were examined in three tier 3 (specialist or highly specialist) CAMHS clinics across two Trusts: Nottinghamshire Healthcare NHS Trust (NHCT) and Derbyshire Healthcare NHS Foundation Trust (DHCFT). Two clinics participated in Nottinghamshire, and one in southern Derbyshire.

Case-notes were accessed by a member of the Trust’s clinical team. NHCT originally signed up to the CORC scheme but unsubscribed in 2012. The Trust introduced a Commissioning for Quality and Innovation (CQUIN) target in April 2011 to complete any one of the following four measures on the electronic record system: SDQ, HoNOSCA, C-GAS and GBO at time 1 (initial assessment) and time 2 (6 month follow-up, or discharge if sooner)*.* CQUIN targets allow commissioners to reward excellent performance by linking the healthcare providers’ income to the achievement of local improvement goals. DHCFT also signed up to the Consortium scheme several years ago and were in the first wave of the CYP- IAPT provider organisations in 2011/12, but have no specific commissioning targets relating to the completion of outcome measures.

The original audit was conducted between December 2010 and January 2011 with the re-audit taking place between December 2012 and February 2013. The same method and protocol were used for both audits. For the original audit, 20 case notes were examined in Derbyshire and North Nottinghamshire, and 21 in South Nottinghamshire. For the re-audit, 20 case notes were included from each clinic that participated in the original audit. In Nottinghamshire (north and south) there were 1250 open referrals, in southern Derbyshire there were 191.

Ethical approval for the study was granted by the local Research Ethics Committee and Research and Development Departments of NHCT and DHCFT.

### Inclusion criteria

Patient files were selected using a random-number generator. If a given file was absent or excluded the next file along was used. Case notes were included only if they were 'current open cases’, which was defined as having a current referral that had been started within the past 2 years and open for a minimum of 6 months. This time frame meant that all cases should have had the opportunity for completion of Time 2 (follow-up) outcome measures. Referral times were checked via the local electronic patient record system and also through the case notes.

For the original audit, only paper case notes were accessed to determine the use of outcome measures. At this time electronic records were only just being created, as such we would not expect outcome measures completed within this audit period to be entered into an electronic system. Given the advancement of electronic records between the original and re-audit timeframe, both electronic records and paper case notes were viewed to determine the use of outcome measures for the re-audit. In Nottinghamshire, this reflected the local commissioning policy’s CQUIN target requiring Time 1 (T1) and Time 2 (T2) measures to be completed electronically. Unless otherwise stated, measures were considered to be present if they were found in either electronic or paper format.

### Audit tool

An identical audit tool was used to record the presence and frequency of use of each outcome measure for the audit and re-audit. This tool contained a timeline, calendar, and list of the relevant measures, including the CORC core measures of SDQ, HoNOSCA, C-GAS, GBO and CHI-ESQ. For the re-audit we specifically recorded whether the measure was present in electronic form, paper form or both. This was not conducted for the original audit because the option of entering CORC measures to the electronic records system had not been developed at the time.

### Analysis

Where appropriate, Chi-square tests were conducted to investigate differences in the use of each outcome measure between the original and re-audit. Chi-square analysis was conducted separately for each outcome measure, where cells had an expected frequency of less than 5, Fisher’s Exact Probability test was used.

## Results

### Frequency and type of outcome measure

Table 1 shows the frequency and use of measures across the three sites for both audits. Comparison of the two audits reveals that the use of generic measures has increased since 2010–2011 (the period of the original audit). For both audits, measures recommended by the Consortium were more likely to be used, with HoNOSCA being the most frequently reported, followed by C-GAS. Comparison of the original and re-audit showed there was a significant increase in the use of these two measures between the two time points (see Table 1). The SDQ parent and self-reported versions were more likely to be completed than the teacher version, and use of parent and self-reported SDQ had significantly increased since the original audit.

**Table 1:** Frequency of the single use of assessment and outcome measures across CAMHS

**Name of measure**	**Total across 3 sites (%)**		**Chi-square/Fisher’s exact**
			*p **< .05**
	**Original audit**	**Re-audit**	
	***n *** **=61 (%)**	***n *** **=60 (%)**	
HoNOSCA	42 (69)	56 (93)	*X*^*2*^ = 28.87, *p* = .001*
SDQ-P	19 (31)	36 (60)	*X*^*2*^ = 10.16, *p* = .001*
SDQ-T	8 (13)	4 (7)	*X*^*2*^ = 1.41, *p* = .363
SDQ-S	14 (23)	38 (63)	*X*^*2*^ = 20.13 *p* = .001*
C-GAS	25 (41)	45 (75)	*X*^*2*^ = 14.35 *p* = .001*
GBO	0 (0)	2 (3)	Fisher’s exact sig. = .244
CHI-ESQ	0 (0)	1 (2)	Fisher’s exact sig. = .496
Conners’ – Teacher	11 (18)	1 (2)	*X*^*2*^ = 9.07, *p* = .003*
Conners’ – Parent	11 (18)	2 (3)	*X*^*2*^ = 6.82, *p* = .009*
RCADS	0 (0)	3 (5)	Fisher’s exact sig. = .119

HoNOSCA, Health of the Nation Outcome Scales for Children and Adolescents; SDQ Strengths and Difficulties Questionnaire (P = parent, T = teacher, S = self); GBO, Goals Based Outcome, C-GAS, Children’s Global Assessment Scale; CHI-ESQ, Commission for Health Improvement-Experience of Service Questionnaire; RCADS, Revised Child Anxiety and Depression Scale. NHT, Nottinghamshire Healthcare NHS Trust, DHCFT, Derbyshire Healthcare NHS Foundation Trust. *p < .01.

Of the other Consortium measures, little evidence was seen of the use of the GBO and CHI-ESQ in both audits. Whereas the Conners’ scale (a standard ADHD assessment scale) [21] was the most frequently used condition-specific measure in the original audit, use of these scales has significantly decreased in favour of more generic measures. Instead, the re-audit revealed that the RCADS was the most frequently occurring condition-specific measure, although this was limited to site three which recently began the introduction of the CYP-IAPT suite of outcome measures.

The re-audit revealed that clinicians were using a greater variety of measures than in 2009–2011. The original audit revealed that 48% (29/61) of case-notes included 1 or 2 different measures, with only 36% (22/61) using 3–5 different measures. In comparison, the re-audit showed that 23% (14/60) of case-notes included 1 or 2 measures, but most (72%, 43/60) included 3–5 different measures. Additionally, in the re-audit, 8% of case-notes included more than 5 different measures. Whereas 16% (10**/**61) of case-notes in the original audit contained no evidence of any measures, this had dropped to 3% (2/60) in the re-audit.

### Repeated use of measures

The repeated use of the same measure had increased from the original audit; however repeated use was still low. The original audit found that only 30% (18/61) of case-notes contained at least one repeated outcome measure. For the re-audit 60% (36/60) of case-notes contained at least one repeated measures, of which only 6% (4/60) had repeated use of *all* their selected baseline measures.

The original audit found that the generic measures were unlikely to be used more than once, with only HoNOSCA being repeated in 10% (6/61) of cases. The re-audit found a significant increase in repeated instances of HoNOSCA, with 50% (30/60) of cases (*X*^*2*^ = 23.35, *p* = .001) containing more than one use of this measure. Similarly, whereas no evidence of repeated use of C-GAS was found in the original audit, 39% (23/60) of cases showed repeated use of this measure in the re-audit (*X*^*2*^ = 28.87, *p* = .001).

There was no evidence of repeated use of the SDQ in the original audit, and although this had significantly increased by the re-audit (Fisher’s Exact sig. = .002), only 17% (10/60) of case-notes in the re-audit had repeated SDQs. Repeated use of GBO was found in 6% (3/60) of cases in the re-audit, and no cases in the original audit.

Whereas the condition-specific Conners’ scale was repeated in 7% (4/61) of case-notes in the original audit, there was no evidence of repeated condition-specific scales (Conners’ or RCADS) in the re-audit.

### Electronic records

The electronic record systems in both Trusts allowed entries of all CORC core measures. As the option for entering CORC measures on an electronic system had not been developed in 2010–2011, information regarding electronic records of measures is available for the re-audit only. Here, measures were slightly more likely to be recorded on paper than electronically, however the difference was small. HoNOSCA was present in electronic records for 77% (46/60) of cases and in paper form for 80% (48/60). C-GAS showed the most marked difference between electronic and paper records with only 47% (28/60) being recorded electronically in comparison to 73% (44/60) on paper. The parent SDQs were found on electronic records in 42% (25/60) of cases and on paper in 55% (33/60) of cases. Self-completed SDQs were found on electric records in 50% of cases (30/60) and on paper in 42 (37%) of cases. There was no evidence of teacher completed SDQs on electronic record and only 7% (4/60) of paper case notes contained this measure.

Across all sites HoNOSCA was the most likely measure to be completed more than once in electronic records (45%, 18/40) and within the stipulated time frame (30%, 12/40), this was followed by C-GAS (repeated use = 33%, 13/40; within time frame = 18%, 7/40). SDQ’s were the least likely to be used more than once (10/40; 25%) and least likely to be completed within the time frame (4/40; 11%). There were no repeated GBOs in electronic records.

### Combined use of outcome measures

In the original audit 14 case-notes (23%) contained a single outcome measure, of which HoNOSCA was used in 10 of these cases (77%), CGAS in 3 cases (5%), and SDQ in 1 case (8%). The use of a single type of outcome measure was only present in 3 (5%) cases for the re-audit, of which one case used HoNOSCA, one an SDQ and one a Conners’.

**Figure 1:**
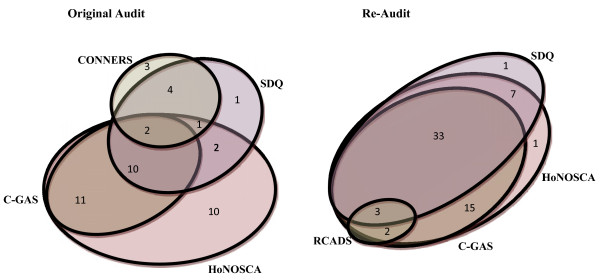
**Overlap of assessment and outcome measures observed in the original audit and the re-audit across three CAMHS sites.** For clarity, different respondents for the SDQ (parent, teacher, self) and Conners (parent and teacher) are combined. Numbers located in each overlapped area indicate the number of case-notes in which the combination of these measures was applied.

Figure 1 illustrates that there was an increase in the combined use of measures from the original audit to the re-audit. Whereas HoNOSCA and C-GAS (18%, 11/61) was the most commonly occurring combination of measures in the original audit, HoNOSCA, SDQ and C-GAS was the most common combination in the re-audit (55%, 33/60). No cases in either the original or re-audit contained the full suite of CORC measures (HoNOSCA, SDQ, C-GAS, GBO and CHI-ESQ). However, one case in the re-audit contained the original CORC measures of HoNOSCA, SDQ, C-GAS and CHI-ESQ. The use of the GBO was only found in one case, and had been used in conjunction with HoNOSCA, SDQ and C-GAS.

In the original audit, the Conners’ scales were the most frequently used condition-specific scale, but the RCADS was the most frequently observed condition-specific measure in the re-audit.

## Discussion

We compared the use of ROM in CAMHS from an audit conducted in 2011 [13] with a re-audit conducted in 2012/2013 to assess for any changes in the use of outcome measures as a result of recent research, government and commissioning strategies. Our findings reveal a significantly greater uptake of HoNOSCA, SDQ and C-GAS, which are measures advocated by a national Child and adolescent mental health services Outcome Research Consortium (CORC), and crucial to fulfil targets outlined in local commissioning policy (CQUIN). We also noted an increase in the repeated use of outcome measures.

In line with the findings from the original audit [13], HoNOSCA was the most frequently used measure in the re-audit, followed by C-GAS. Additionally, both the single and repeated use of these measures had significantly increased since the original audit. As reported by Batty et al. [13], HoNOSCA has a longstanding history and expectation of use within these Trusts which is likely to explain its high completion rate. As both HoNOSCA and C-GAS are clinician-completed measures it is easier for clinicians to ensure that these measures are completed, in comparison to measures completed by the service user or caregiver (e.g. SDQ, GBO, CHI-ESQ) which often involve more administrative support and the co-operation of the service user. The improved completion rates for clinician rated outcome measures offer the service user, clinician and managers with quantifiable evidence of any change that may resulted from the intervention. This allows all stakeholders to assess the effectiveness of the service and the individual may assess the benefit of the treatment received. However, the comparatively lower completion of service user measures demonstrates that the perspective of change as recorded by the service user is under-reported. It is important that change from both the perspective of the clinician and service user is recorded to fully understand the effectiveness of any intervention. This is particularly the case as clinician completed measures can be susceptible to reporting bias, such as over reporting the extent of improvement [22].

The re-audit revealed a greater use of combined measures in comparison to that found in the original audit. As shown in Figure 1, in the original audit HoNOSCA and C-GAS were the most common combination of measures, whereas in the re-audit, HoNOSCA, SDQ, and C-GAS were the most common grouping of measures, with this combination appearing in over half of all case notes. This suggests that clinicians are in greater agreement regarding which combination of outcome measures to use. The increased uptake of the SDQ may also reflect clinicians’ positive attitudes towards this measure, with previous research showing that they value this as a measure of service users’ opinion [13]. Additionally, the increase in provision of administration time for outcome measures may have been influential for the completion of these service-user completed measures which involve extra burden in terms of posting-out and collecting questionnaires. In contrast, other CORC advocated measures such as the GBO and CHI-ESQ were rarely found. Again, it may be that this represents difficulties in getting measures completed by the service user or, given that these measures are relatively new with less established psychometric properties and history of use within CAMHS, clinicians may be unwilling to engage with these measures. Previous research has noted concerns about the scientific quality of measures and lack of knowledge in how to use and interpret a measure impedes clinicians’ likelihood to use a measure [11,14,23]. It is also possible that clinicians feel that a combination of the clinician completed measures alongside one service user measure, such as the SDQ which has established validity and reliability [24-26], is satisfactory in gaining an impression of current functioning.

The significant increase in both the single and repeated use of different outcome measures since the original audit may have resulted from factors other than increased administration time. Additionally, initiatives such as CORC have promoted greater awareness or the use and type of outcome measures, specifically, in the use of generic rather than condition-specific measures that allow comparisons to benchmarks which should lead to improvements in practice [27]. The influence of the recent IAPT initiative is also already seen in DHCFT, whereby the uptake of the IAPT advocated RCADS was found in some case notes. Although routine use of all CYP-IAPT scales was only introduced after the re-audit, the initial year of the scheme involved training clinicians on the clinical usefulness and importance of completing ROMs, thus establishing a managerial expectation that they would be completed. However, it is interesting to note that NHCT withdrew their CORC membership after the original audit but increased their use of outcome measures, suggesting that any practical support provided by CORC was less influential.

We have also speculated that the collection of outcome measures may be driven by CQUIN targets, but DHCFT did not have any commissioning strategies associated with the completion of outcome measures (i.e. CAMHS outcomes CQUIN); yet comparable results were found across the sites with regard to CORC measures. Thus, it may be that clinicians’ willingness to use a measure may be driven as much by knowledge and awareness of measures as it is by commissioning policies. Since the original audit, the research organisation, Collaborations for Leadership in Applied Health Research and Care – Nottinghamshire, Derbyshire, Lincolnshire (CLAHRC-NDL) has conducted significant work to promote the use of outcome measures across the East Midlands. Such work includes seconding 'Diffusion Fellows’ and other local champions from NHS partners to translate and disseminate knowledge from research studies into practice, holding conferences and seminars for local clinicians and publishing findings of the original audit in simple summary 'bites’ for clinicians and managers. Given that previous research has highlighted local champions as providing a key role in promoting outcome measures [28], it is likely that CLAHRC activities have also partially driven this change across the two NHS Trusts.

Whereas the original audit [13] and previous research [11] noted very little repeated use of the same measure, we found that approximately 60% of case-notes contained repeated use of the same measure, compared to 30% in the original audit. Although this shows an improvement in ROM, given that almost half the case-notes still contained only a single use of a given measure, there is further work needed to improve the rate of outcome measurement in CAMHS.

As the option of entering CORC measures to the electronic records system had not been developed at the time of the first audit, we cannot make comparisons regarding the number of measures that are being recorded electronically. However, findings from the re-audit demonstrate that the majority of measures are being recorded electronically. This is important for a care system where multiple professionals with specialised knowledge may be involved in care delivery in different geographical areas [29]. It is possible that the option of electronic inputting of data may have contributed to the increased use of outcome measures, allowing clinicians to quickly input clinician-completed outcome measures (HoNOSCA and C-GAS) without having to find paper-versions. Electronic records offer the opportunity of better access to patient information, with the premise that the greater the availability of high quality information the more able the clinician is to care for the patient [30]. Furthermore, the use of an electronic system allows opportunity for the development of a report system that graphically represents changes in outcome scores over time. This system would provide clinicians with real-time feedback on their client’s progress, thus increasing the clinical utility of outcome measures [13]. Additionally, this type of report could allow data to be aggregated at team or service level to inform managers and commissioners to enable benchmarking to comparable services [31].

Although the re-audit has shown there is a significant increase in the uptake of ROM in child and adolescent psychiatry, it has also highlighted the need for further improvement, particularly with regard to repeated use of the same measure and measures completed by the service user (e.g. SDQ, GBO, CHI-ESQ). In order for an outcome measure to assess any changes that may have resulted through an intervention it is imperative that the same measure is used at baseline and at least once thereafter. To reduce burden on clinicians future outcome measure initiatives may wish to consider reducing the number of clinician completed measures required. Given that HoNOSCA has been shown to have better reliability and be more informative than C-GAS [32] it may be prudent to only complete HoNOSCA. However, C-GAS provides information about the level of functioning of the service user in the previous month [4] across all conditions, incorporating elements of a multi-axial assessment [33]; therefore it is considered a valuable complement to HoNOSCA in research [34] and clinical practice. To improve the completion of service user completed outcome measures such as the SDQ, new technologies could be implemented to facilitate their use. For example, the measures could be completed on a tablet PC in the waiting room prior to each clinic session, (a system currently being rolled out by the CYP-IAPT initiative and separately a trial of electronically completed measures which is being evaluated by the CLAHRC-NDL). The reports from these measures could be fed straight back to the clinician online, producing real-time feedback that does not rely on service users having to remember to complete and post questionnaires prior to their clinic appointment. Truman et al. [35] report on a computer-based SDQ and found significantly more user satisfaction with a computer version in comparison to the paper-based version. This kind of measure requires significant investment in technical adaptation to ensure integration with electronic patient records and would require managerial understanding and commitment to proceed. This 'session-by-session’ monitoring would overcome difficulties in getting follow-up measures due to treatment drop-out, or clinic appointments that are not scheduled around the 6-month follow-up. Furthermore, this regular monitoring may be more sensitive to change and may also allow clinicians to modify their intervention strategy earlier on if they felt sufficient progress was not being made [36].

The comparison of two audits has offered a valuable insight into the improvements of ROM within child and adolescent psychiatry which may have resulted from greater Trust support and initiatives such as CLAHRC-NDL research, CORC and CYP-IAPT that actively promote the use of outcome measures. However, our findings are limited to two NHS Trusts; as such caution should be taken when generalising the findings to other Trusts located in different geographical regions. Nethertheless, the comparison of two different Trusts allowed for the assessment of local service drivers and priorities and their impact on outcome measure completion. Given that the aim of this research was to document the evidence-base for ROM in CAMHS we did not assess clinicians’ opinions as to which factors were influencing their use of specific outcome measures in the re-audit. However, we have inferred possible barriers based on well documented findings from previous research [13,14,16,17,20].

## Conclusion

A comparison of two audits has revealed an increase in the use of outcome measures within CAMHS, particularly for clinician-completed measures. The possible increase in clinician awareness and training in outcome measures, alongside dedicated administration support may have facilitated the process of ROM. It is important that initiatives continue to increase clinicians’ awareness of the importance of measuring outcome within CAMHS, with education specifically focussing on the need to assess whether patients have benefited from the result of the intervention, in line with more physically-based health services. Further initiatives are also needed to improve the repeated use of measures and service user completed measures. As described, innovative models of session-by-session monitoring on tablet PCs could further increase clinician and service-user engagement with outcome measures by providing instant, clinically-useful feedback.

### Ethical & R & D approval

Ethical approval was granted by the Nottinghamshire Ethics Committee and R&D approval was obtained from the two NHS Trusts (Nottinghamshire Healthcare NHS Trust (NHCT) and Derbyshire Healthcare Foundation Trust (DHCFT).

## Competing interests

The authors declare that they have no competing interests.

## Authors’ contributions

MM and, LB collected the data from the case notes. CLH oversaw data collection and analysed the results. All authors contributed to the interpretation of the data and the study write-up. CLH drafted the manuscript and MM, LB, MMa, KN, JT, KS and CH revised it critically for important intellectual content. All authors read and approved the final manuscript.

## Pre-publication history

The pre-publication history for this paper can be accessed here:

http://www.biomedcentral.com/1471-244X/13/270/prepub
